# Does the use of EndoFLIP during fundoplications improve outcomes?

**DOI:** 10.1007/s00464-025-11840-z

**Published:** 2025-06-09

**Authors:** Andrew F. Sabour, Emily D. Duckworth, Marvin A. Rhodes, Dawn W. Blackhurst, Erica Schumann, Shanu N. Kothari, Brian Hodgens

**Affiliations:** Division of Surgery, Department of Surgery, Prisma Health/USC School of Medicine Greenville, 905 Verdae Blvd, Suit #202, Greenville, SC 29607 US

**Keywords:** Foregut, Hiatal hernia, Fundoplication, EndoFLIP, Planimetry, Distensibility

## Abstract

**Background:**

Impedance planimetry (EndoFLIP) provides a powerful intraoperative tool to the trained Foregut surgeon. Real time data on esophageal diameter and distensibility index (DI) allows for objective biofeedback to improve the overall consistency of the operation. While EndoFLIP carries great potential, little has been said about its effect toward postoperative outcomes. Our goal for this study was to identify how certain EndoFLIP scores would impact postoperative patient surveys.

**Methods:**

A prospectively collected database of patients undergoing crural repair between January 2023 and September 2024 was reviewed. EndoFLIP measurements of DI and diameter were obtained after fundoplication using a standardized protocol. Patients were then surveyed postoperatively at 2 months, 6 months, and 1-year intervals to trend changes in GERD-HRQL, REGURG, LPR-RSI, and Dysphagia scores. Spearman Rank correlation was used to assess linear relationships between post-fundoplication measurements and quality-of-life scores at follow up.

**Results:**

A total of 55 patients were reviewed in this study. All patients underwent a crural repair while 45% underwent an anterior wrap and 55% a posterior wrap. Diameter and DI measurements were not found to correlate with GERD-HRQL or LPR-RSI scores at 1-year postoperative visits. No correlation was also found when looking at the change in scores from preoperative to postoperative surveys. Larger DI and diameters did correlate to higher REGURG scores at 1-year follow ups.

**Conclusions:**

Intraoperative DI and diameter measurements carry almost no correlation to postoperative outcome scores. Only a positive correlation to REGURG scores with higher DI and diameter were noted. As long as DI is kept between extreme values, other factors must be investigated as part of a multifactorial influence to postoperative dysphagia and GERD.

A paraesophageal hernia repair with fundoplication remains the gold standard for surgical treatment of reflux disease. Classically, intraoperative decision-making regarding fundoplication creation relied on visual and tactile feedback, leading to significant variability in surgical outcomes within the community. Introduction of the functional lumen imaging probe (EndoFLIP) allows surgeons to obtain real-time, objective measurements to guide intraoperative adjustments [1]. EndoFLIP provides quantifiable data that helps standardize surgical approaches and improve the consistency of postoperative outcomes. It is imperative to understand the function, values, and capabilities of the EndoFLIP to provide the best future outcomes for our patients.

First available for commercial use in 2009, the EndoFLIP uses an inflatable catheter to identify the diameter and distensibility index (DI) of the esophagogastric junction (EGJ). Early studies looked at asymptomatic patients, either randomly selected or during unrelated laparoscopic procedures, to identify normal DI values [2, 3]. In 2010, Kwiatek et al. compared GERD patients with a group of controls and found that reflux patients exhibited a DI 2 to 3 times higher than normal [4]. The same study also found that an estimation of the EGJ on endoscopy correlated poorly to the actual DI value. These findings established two key ideas. First, DI provided a quantifiable, physiologic difference between patients with and without reflux. Second, EndoFLIP allowed a reliable, repeatable method of assessing the true distensibility of the EGJ.

Modifying a fundoplication based on EndoFLIP measurements may allow for consistent and improved postoperative outcomes [5]. An EGJ with a post-fundoplication DI (FDI) > 4.5 can indicate that the repair is too loose, leading to higher rates of recurrence [6]. Conversely, a low FDI may show that a wrap is too tight, increasing the risk for postoperative dysphagia. In a study by Su et al., patients with an FDI < 2 reported worse gas bloat and dysphagia at both 1 year and 2 years postoperatively [7]. These findings led to the Northshore group recommending a final FDI range between 2 and 3.5 mm^2^/mm Hg [5]. Unfortunately, the recommended FDI ranges to avoid dysphagia have varied widely among different institutions. Several studies now find that an FDI < 0.5 would be a more appropriate lower limit, with patients in these populations oftentimes finishing with an FDI < 2 and having no changes to their dysphagia [8–11]. The stark contrast in numbers raises concern about the method used to obtain measurements, and whether a more standardized approach is necessary. Perhaps, instead of relying solely on FDI, alternative metrics such as diameter or changes in DI should be used instead [9, 12]. We hypothesize that not only is a lower FDI safe for long-term outcomes, but also that following EndoFLIP measurements intraoperatively can lead to improved postoperative outcomes.

## Materials and methods

### Data collection

Data were collected through a retrospective review of a prospectively maintained database between January 2023 and September 2024. All data were collected at a single institution and included patients undergoing either robotic or laparoscopic paraesophageal hernia repair with fundoplication. Any type of fundoplication was included and categorized as either a partial posterior wrap or a partial anterior wrap. EndoFLIP measurements must have been obtained after hiatal dissection and again after fundoplication. Postoperatively, patients were followed with subjective quality-of-life (QoL) surveys to calculate Gastroesophageal Reflux Disease-Health Related Quality of Life Questionnaire (GERD-HRQL) and Reflux symptom index (RSI) scores. A regurgitation score (REGURG) was calculated by totaling the score of six isolated regurgitation questions from the GERD-HRQL survey. Dysphagia and bloating scores embedded in the GERD-HRQL were individually tracked as well. Patients were also asked whether they continued to use their proton pump inhibitors (PPI) or H2 blockers for symptom control. Surveys were performed in clinic at time intervals of 2 months, 6 months, and 1 year. Only patients who consistently followed up at all three time intervals were included in the final analysis. Institutional review board approval was obtained for this study.

### Operative protocol

Once patients completed their preoperative work up and deemed fit for surgery, they were scheduled for surgery with plans for an overnight stay. Fundoplication selection was based on a combination of preoperative work up, intraoperative measurements, surgeon preference, and patient symptoms. Patients were positioned in 20 degrees of reverse Trendelenburg at the beginning of each case. Abdominal entry was performed using a direct Optiview trocar just left of the umbilicus. A Nathanson liver retractor was used for all cases.

Once visualization was achieved, the hernia was reduced, and the hiatal dissection began in a regimented approach starting from the right crus. After complete circumferential dissection of the hiatus, esophageal mobilization was performed until a goal of at least 3 cm of intrabdominal esophagus was achieved. Positioning of the lower esophageal sphincter (LES) was always confirmed on endoscopy. Care was always made to identify both vagal nerves throughout the process. EndoFLIP measurements were obtained after esophageal mobilization but prior to crural repair. The hiatus was then reapproximated using permanent suture, no mesh, in an interrupted fashion.

When performing the fundoplication, care was made to ensure that the LES was always included. A partial posterior wrap had the fundus wrapped posterior to the esophagus and tacked anteriorly in two columns of three interrupted, permanent sutures. Partial anterior wraps had the fundus wrapped anterior to the esophagus and tacked to both the left and right crus through a set of interrupted, permanent sutures. A bougie was never used during the fundoplication. EndoFLIP measurements were obtained immediately after fundoplication. An endoscopy was then performed after the EndoFLIP to ensure proper location of the fundoplication at the GEJ and a visually appropriate flap valve prior to completion of the procedure. Patients were kept overnight on a full liquid diet and typically discharged the next day.

### EndoFLIP system and protocol

Intraoperative measurements were obtained using the EndoFLIP EF-325 model. This model utilizes an 8 cm balloon catheter with 17 impedance planimetry ring electrodes each 5 mm apart. For our study, we obtained measurements by filling the catheter balloon using 40 and 50 cc of fluid. A fill of 40 cc was picked to follow a formal standardized protocol by Su et al. in obtaining consistent, optimized measurements [13]. We also included a 50 cc fill to add data for a rarely used setting. All patients undergoing EndoFLIP were paralyzed under general anesthesia. Pneumoperitoneum was removed prior to each set of measurements. Patients were kept in a reverse Trendelenburg position and with a liver retractor in place.

Upon initial use, the EndoFLIP catheter was placed transorally into the stomach under direct laparoscopic visualization. Once gastric placement was confirmed, the abdomen was completely desufflated. The catheter was then filled to 30 cc and gently pulled back until the desired hourglass configuration was obtained. Measurements were then recorded after waiting a total of 30 s. The balloon fill was then increased to 40 cc and then 50 cc and repeat sets of measurements were taken after 30 s each. At completion, the catheter balloon was emptied and withdrawn back into the esophagus. If the post-crural or post-fundoplication DI was found to be < 0.5, steps were taken to loosen the repair to reduce the risk of postoperative dysphagia [8, 10, 14]. Patients with a high DI were approached on a case by case basis, but no specific barrier was placed.

### Statistical analysis

Post-fundoplication measurements are presented as medians and interquartile range (25th and 75th percentiles). The Wilcoxon Rank Sum test was used to test differences between 2 groups. Spearman Rank correlation was used to assess linear relationships between post-fundoplication measurements and QOL scores at follow up. All statistical analyses were completed using SAS statistical software (SAS Enterprise Guide 8.3, Cary, NC). *P*-values < 0.05 were considered indicative of statistical significance.

## Results

### Patient demographics

A total of 136 patients were initially included within the parameters of our study. Of these patients, 55 (40.4%) maintained serial follow ups up to 1 year postoperatively. Patients with 1-year postoperative follow ups had a median age of 69 years and averaged 78% female with a median BMI of 28 (range 18 to 37).

### Fundoplication types

An even distribution of fundoplications were performed within the study (25 anterior vs 30 posterior) (Table 1). When comparing demographics between the two types of fundoplication, neither gender, BMI, nor medical comorbidities were found to be significantly different. Patients undergoing partial anterior fundoplications were significantly older than those receiving partial posterior fundoplications (72 vs 63 years, *p* = 0.002). Initial symptoms and whether the patient had a recurrent hiatal hernia did not influence the type of fundoplication performed. If a patient was on a PPI preoperatively, there was an obvious bias toward performing a posterior rather than anterior fundoplication (93.3% vs. 64%, *p* = 0.015).

**Table 1:**
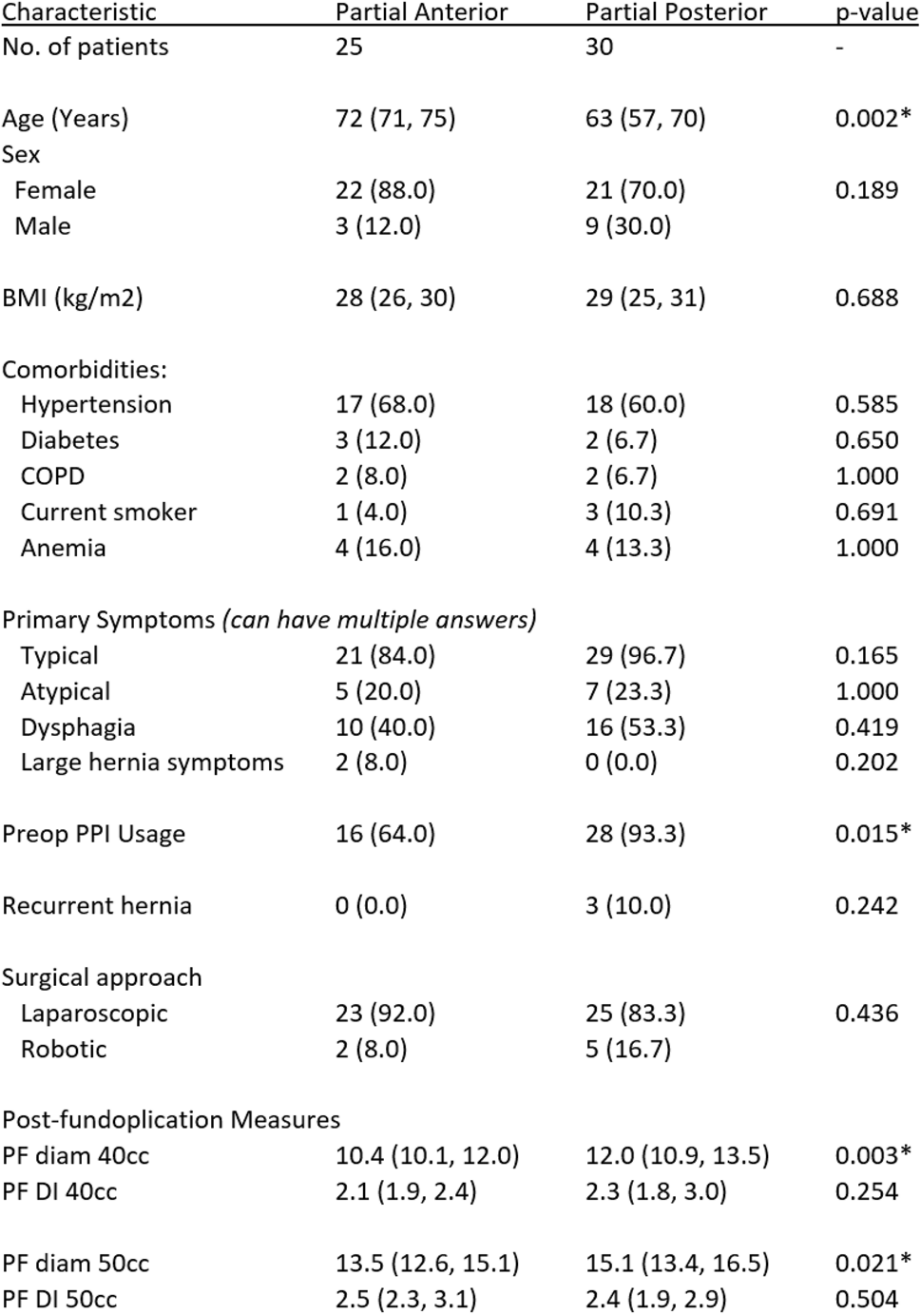
Patient and operative characteristics by type of fundoplication

Intraoperatively, performing the procedure laparoscopic or robotic had no impact on the type of fundoplication a patient received. Post-fundoplication EndoFLIP measurements were also compared between the types of fundoplication performed. While FDI had no significant difference based on the type of fundoplication, diameter was significantly smaller in the partial anterior wrap than the posterior wrap for both fills (10.4 vs. 12 at 40 cc, 13.5 vs. 15.1 at 50 cc) (Table 1).

### Overall outcome comparisons

Patient outcome surveys were tabulated against diameter and DI values at 1-year postoperative follow ups. Initial analysis compared outcome scores at 1-year postoperative to FDI and diameter scores. Secondary analysis was then performed looking at the change in preoperative to postoperative outcome scores versus the patients FDI and post-fundoplication diameter. A Spearman rank correlation was calculated to assess for significance.

**Fig. 1:**
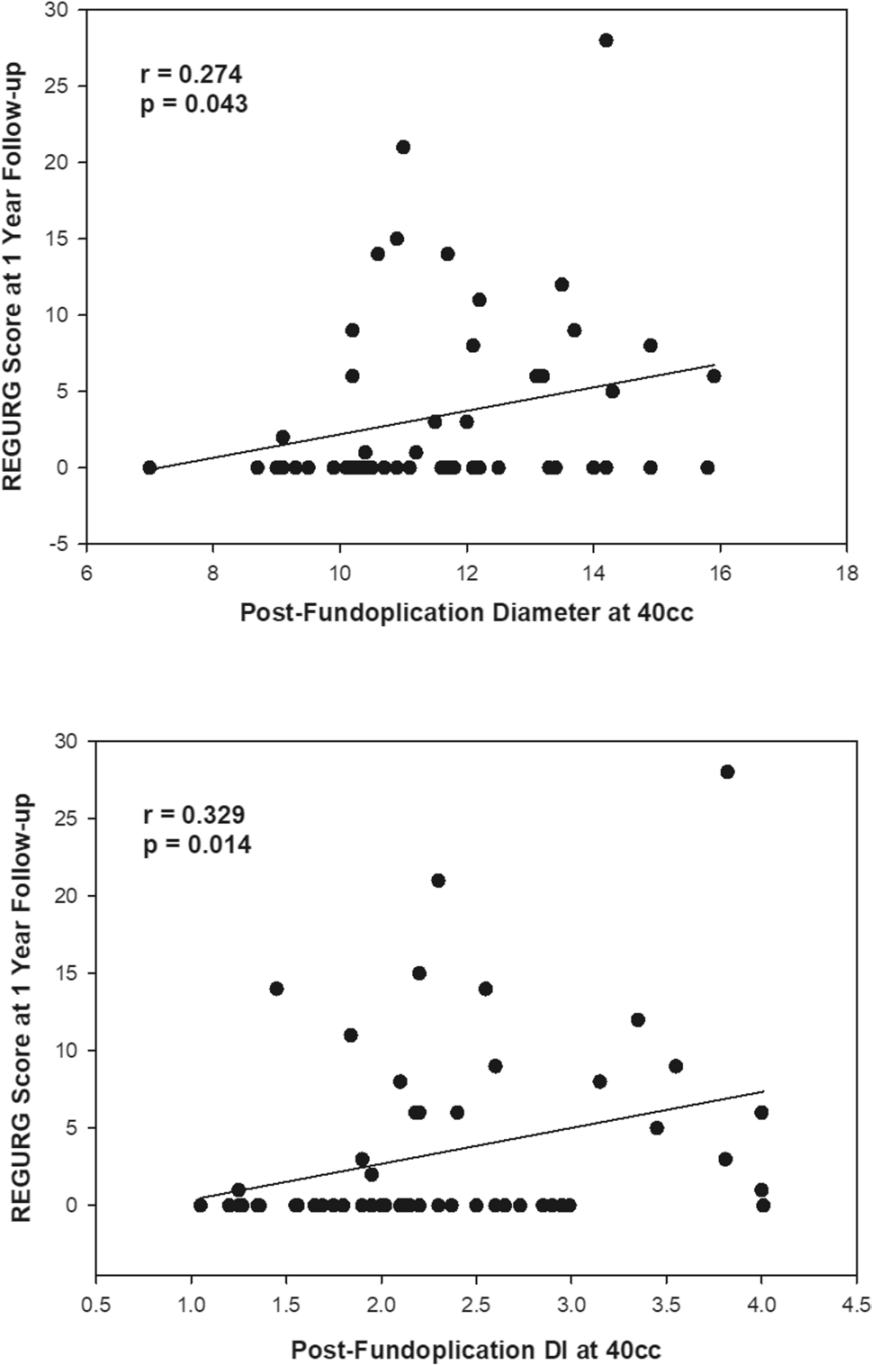
Correlation of post-fundoplication diameter and DI at 40 cc fill with REGURG score at 1-year follow up

When looking at the final values for outcome surveys, only REGURG scores showed a positive linear relationship to FDI and diameter at 40 cc (*p* = 0.043, *p* = 0.014) and FDI at 50 cc (*p* = 0.045) (Fig. 1). No other significant correlation was identified from GERD-HRQL and LPR-RSI surveys (Table 2). However, when looking at the change in scores from baseline to 1-year postoperative outcomes, REGURG scores kept a positive linear relationship but lost their statistical significance. No other survey scores demonstrated any correlation to intraoperative DI or diameter.

**Table 2:**
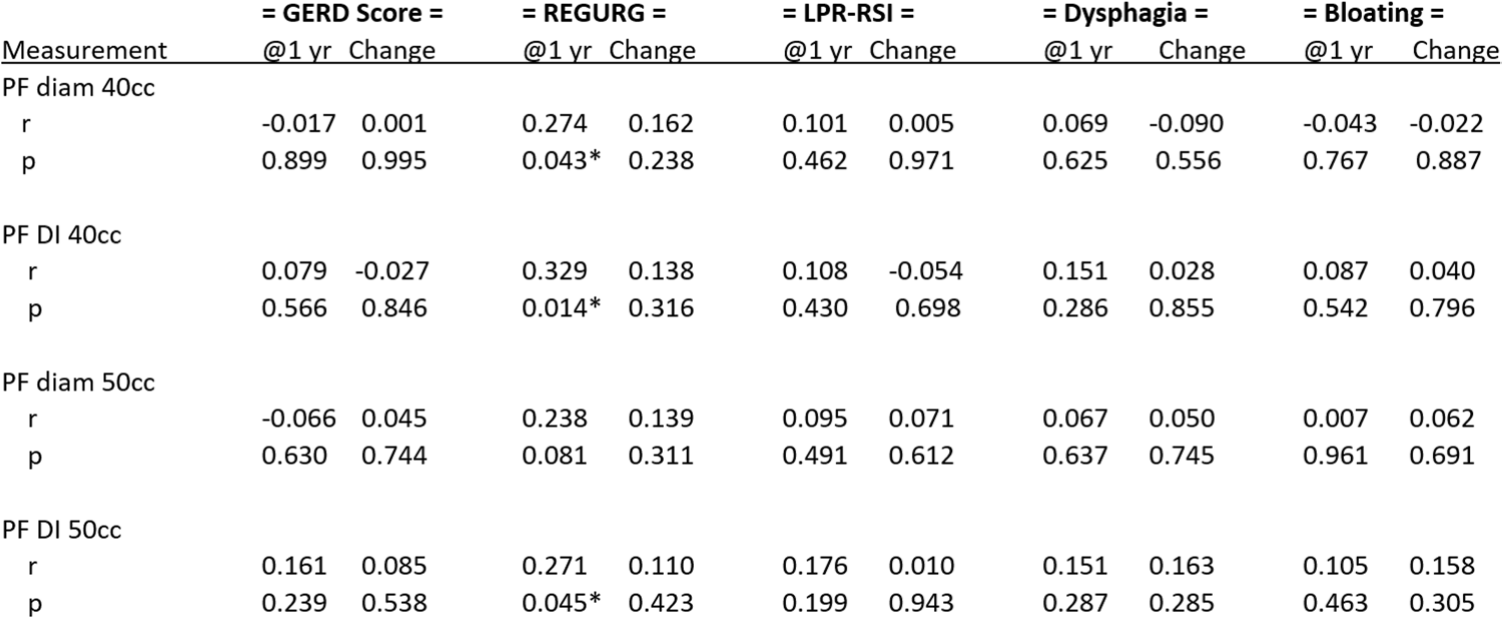
Correlation of post-fundoplication measurements (diameter and DI) with symptom QoL scores at 1 year and change from baseline at 1 year

### PPI/H2 blocker usage

Besides survey scores, our group asked patients at each postoperative interval whether they took their PPI or H2 blocker for symptoms control. Patients were grouped into “yes” or “no” takers and evaluated based on their average DI and diameter at 40 and 50 cc. In total, 40 (72.7%) patients were not on PPI or H2 blockers on the 1-year postoperative visit after reflux surgery. EndoFLIP measurements were not found to have any statistical significance in determining whether a patient remained or restarted on PPI postoperatively (Table 3).

**Table 3:**
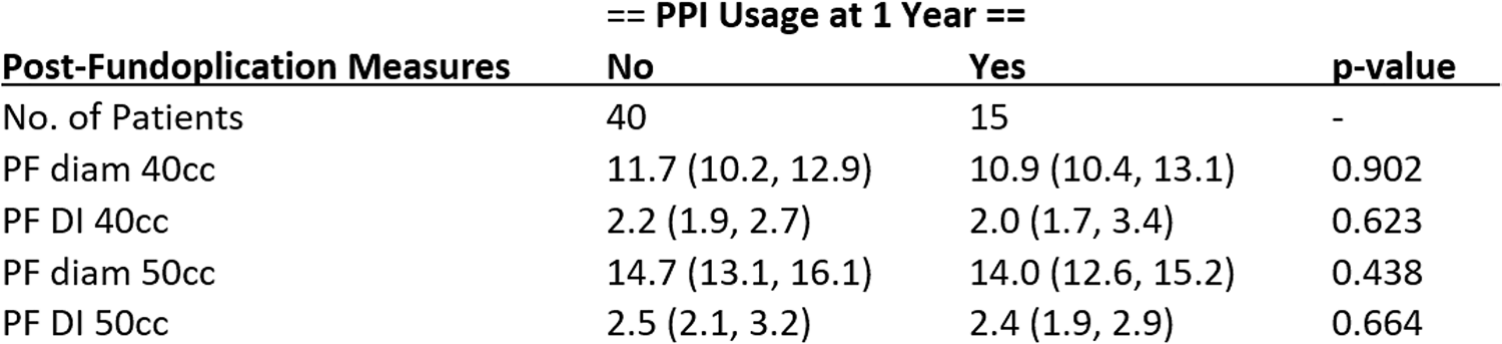
Post-fundoplication measurements (diameter and DI) by PPI usage at 1 year follow up

### Dysphagia/bloating scores

A key clinical question for our study was whether intraoperative EndoFLIP values correlated to postoperative dysphagia or bloating. Both scores were taken at each postoperative interval, and patients were grouped into either “no” with a score of 0–2 or “yes” with a score of 3–5. In general, patients who exhibited bloating had lower diameter and DI values at both balloon fills, although no statistical significance was ever found (Table 4). Patients with dysphagia however averaged higher DI and diameter values but again showed no signs of statistical significance.

**Table 4:**
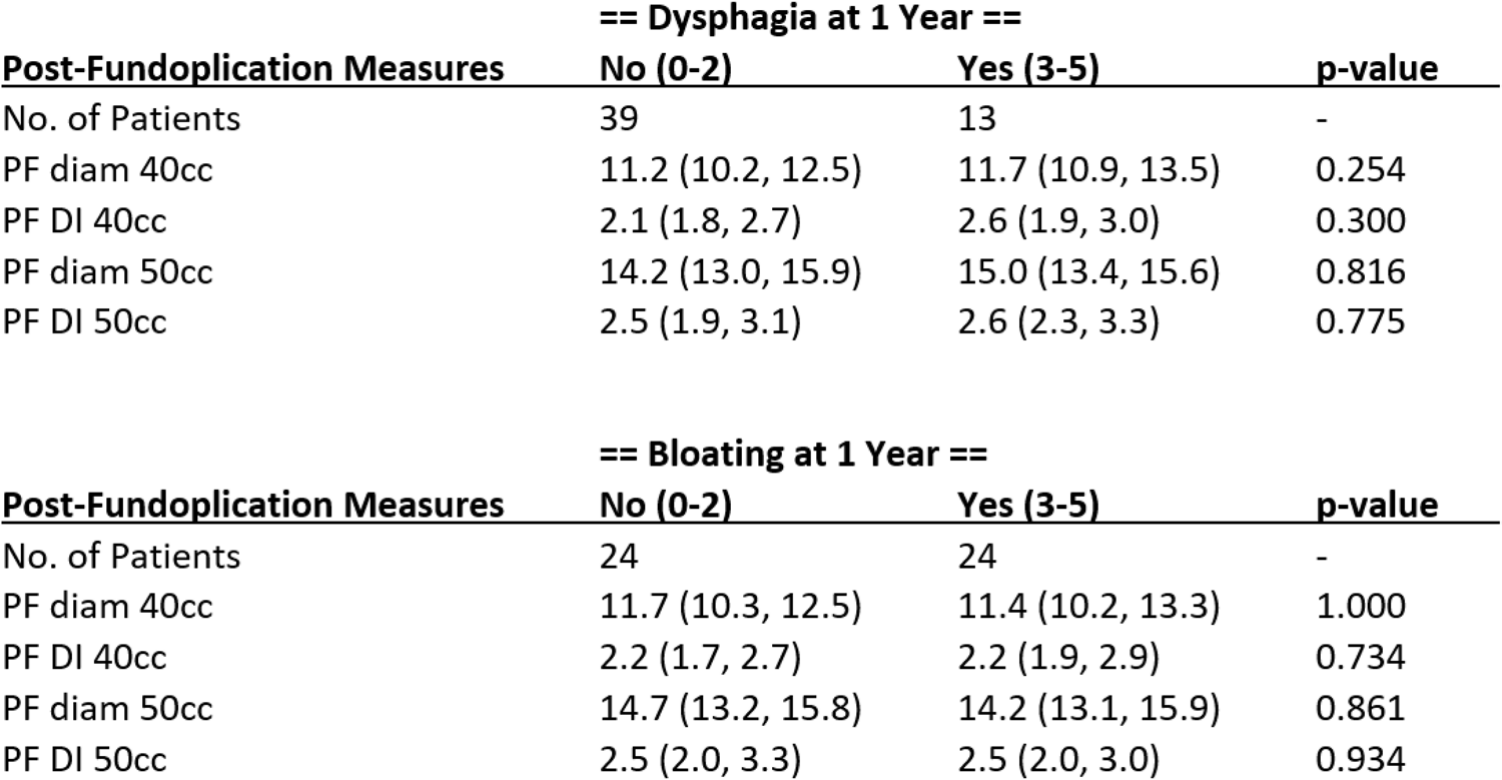
Post-fundoplication measurements (diameter and DI) by presence of bloating and dysphagia at 1 year follow up

## Discussion

The advent of EndoFLIP impedance planimetry has transformed the field of Foregut surgery from an art to a science. With objective intraoperative data, surgeons may now alter their decision-making in hopes of improving postoperative outcomes. Unfortunately, significant variance exists in the device’s implementation and protocol. Components such as patient positioning, use of pneumoperitoneum, size of the balloon (8 or 16 cm), fill of the balloon, the wait time before measurement, and the influence of extraneous factors such recent bougie use, all differ among institutions. Differences in these factors impact the final measurements, leading to trouble when comparing data among different studies. For example, Liu et al. found that pneumoperitoneum makes a difference in DI values, especially when looking at post-crural and fundoplication DI [15]. Yet other studies show no influence of pneumoperitoneum or even patient positioning. The best effort at a standard protocol comes from a series of studies from Northshore, where patients in reverse Trendelenburg had measurements performed without pneumoperitoneum using an 8 cm balloon at 30 and 40 cc of fill [16]. These parameters have slowly integrated into other studies, including ones from this institution.

Given the differences in protocol, direct comparisons between studies remain challenging. However, despite several confounding variables, consensus regarding the impact of extreme measurement values exists. When looking at regurgitation, our study found that higher FDI and EGJ diameter correlated with increased postoperative scores. This aligns with the understanding that trans-EGJ flow depends on EGJ length and diameter, with an increase in diameter creating an easier path for regurgitation [4, 17]. While few studies look at the impact on regurgitation, Amundson et al. recommended an FDI < 6.2 to reduce the risk of hernia recurrence [6]. Others, such as Wu et al., looked at an FDI < 4.5 to best improve postoperative GERD-HRQL and LPR-RSI scores. Ultimately, we recommend modifying any repair with an FDI > 4.5 to reduce the risk of worsening postoperative regurgitation and reflux.

When looking at dysphagia, prior studies emphasized the importance of avoiding a low FDI. Studies from the Northshore group such as Wu et al. remark consistently on using an FDI < 2 as predictor of postoperative dysphagia [7, 16]. Our findings conflict with these results, demonstrating that any DI > 0.5 had no impact on dysphagia up to 1-year postoperatively. In fact, as long as DI was kept between 0.5 and 4.5, we found no impact from either DI or diameter to any type of postoperative outcome score. These results are supported by Amundson et al., who also found no significant differences in EndoFLIP measurements between patients with good or poor postoperative outcomes [6]. Usage of an FDI < 2 does occur throughout the literature with minimal reported outcomes against dysphagia. A study by Ilczyszyn et al. found that out of 17 patients averaging an FDI 1.36, the only patient who demonstrated short-term dysphagia had an FDI of 0.47 [14]. Another study by Al Asadi et al. found that patients undergoing either a Hill procedure or Toupet fundoplication with an FDI < 2 had no differences in dysphagia within 1–2 years postoperatively [11]. While an FDI < 0.5 remains agreed upon as the lowest accepted barrier, the influence of an otherwise low or high DI to dysphagia remains negligible.

With minimal impact of DI and diameter on postoperative outcome scores, there must be a more multifactorial source. Few studies have looked at EndoFLIP measurements besides FDI. Of these, a study by Shah et al. found that the change in DI between a 30 cc and 40 cc balloon fill correlated well to postoperative dysphagia [12]. Patients who had a decrease or no change in DI after an increase in balloon fill had significantly lower postoperative dysphagia scores. Like us, they found that the absolute values of FDI had no correlation to postoperative dysphagia scores. Perhaps instead of the absolute value DI or diameter, the change in value from baseline to post-fundoplication would demonstrate a more significant impact on what the patient feels. Besides DI, Wu et al. demonstrated that EGJ compliance may provide supplemental data to DI in determining postoperative impact [18]. Little else has been done to look at the impact of EndoFLIP measurements and postoperative outcome scores, leaving room for serious further investigation.

### Limitations

Despite reporting a moderate 1-year follow up rate, the primary limitation of our study is a small sample size. Due to the low powered nature of the study, subtle connections between EndoFLIP measurements and outcome scores may have been missed. We hope that in future, a larger powered study with a standardized, easily replicable protocol may conclusively determine significant correlations between EndoFLIP measurements and patient outcomes. A loss in follow up also creates the potential for selection bias, filtering out patients who may have felt disinterested in further visits due to the resolution of their symptoms. Additional efforts should be made to increase follow up rates, possibly prioritizing virtual visits as a quick and simple method of obtaining survey results. Another limitation is the subjective nature of our outcome scores and the lack of objective data such as imaging or endoscopy to help identify hernia recurrence, esophagitis, or fundoplication disruption. Finally, our study suffers from the restriction of a single center design, introducing regional biases such as dietary and lifestyle factors that may differ across patient populations. Due to the vast majority of reflux surgery being elective, the study also suffers from an inherent disparity against patients from a lower financial class or uninsured. This issue may be corrected in future by expanding the study to a multicenter trial with multivariate analysis.

## Conclusion

EndoFLIP remains a valuable intraoperative tool for providing real-time, quantitative assessment of the EGJ. Our study suggests that while higher DI and diameter values may correlate with increased postoperative regurgitation, no clear association exists between EndoFLIP measurements and postoperative dysphagia or bloating. Avoidance of an extremely low DI (< 0.5) or high DI (> 4.5) remains the only clear guidance. These findings highlight the complexity of fundoplication outcomes and suggest that a multifactorial approach, rather than a strict reliance on DI thresholds, is necessary to optimize surgical results.
